# Corrosion Degree Evaluation of Polymer Anti-Corrosive Oil Well Cement under an Acidic Geological Environment Using an Artificial Neural Network

**DOI:** 10.3390/polym15224441

**Published:** 2023-11-17

**Authors:** Jun Zhao, Rongyao Chen, Shikang Liu, Shanshan Zhou, Mingbiao Xu, Feixu Dai

**Affiliations:** 1 China Oilfield Services Limited, Langfang 065200, China; 2 School of Petroleum Engineering, Yangtze University, Wuhan 430100, China; 3 National Engineering Research Center for Oil and Gas Drilling and Completion Technology, Yangtze University, Wuhan 430100, China; 4 Tarim Oilfield Production Capacity Construction Division, Korla 841000, China

**Keywords:** oil well cement, resin, corrosion, artificial neural network, particle swarm optimization, carbon dioxide

## Abstract

Oil well cement is prone to corrosion and damage in carbon dioxide (CO_2_) acidic gas wells. In order to improve the anti-corrosion ability of oil well cement, polymer resin was used as the anti-corrosion material. The effect of polymer resin on the mechanical and corrosion properties of oil well cement was studied. The corrosion law of polymer anti-corrosion cement in an acidic gas environment was studied. The long-term corrosion degree of polymer anti-corrosion cement was evaluated using an improved neural network model. The cluster particle algorithm (PSO) was used to improve the accuracy of the neural network model. The results indicate that in acidic gas environments, the compressive strength of polymer anti-corrosion cement was reduced under the effect of CO_2_, and the corrosion depth was increased. The R^2^ of the prediction model PSO-BPNN3 is 0.9970, and the test error is 0.0136. When corroded for 365 days at 50 °C and 25 MPa pressure of CO_2_, the corrosion degree of the polymer anti-corrosion cement was 43.6%. The corrosion depth of uncorroded cement stone is 76.69%, which is relatively reduced by 33.09%. The corrosion resistance of cement can be effectively improved by using polymer resin. Using the PSO-BP neural network to evaluate the long-term corrosion changes of polymer anti-corrosion cement under complex acidic gas conditions guides the evaluation of its corrosion resistance.

## 1. Introduction

The cement sheath used to seal the formation is prone to be corroded by acidic gas in oil and gas wells and geologically sealed wells containing acidic gas, leading to strength reduction, structural damage, and cement integrity failure [1,2]. The damage to cement casing will affect the quality of cementing. Even wellbore collapse occurs severely, causing severe economic losses and environmental pollution [3,4]. The corrosion resistance of cement slurry under acidic corrosive gas can be effectively improved by adding anti-corrosion materials to improve the performance of cement slurry.

To study the corrosion of cement, Liaudat et al. [5] studied the corrosion mechanism of cement in a CO_2_ environment and found that the degradation of cement structure and strength is caused by the loss of hydrochemical products in a CO_2_ environment. Kuo et al. [6] studied the changes in oil well cement in corrosive environments and concluded that the reaction between calcium hydroxide and CO_2_ in cement decreases cement strength. Yin et al. [7] studied the corrosion changes in cement and proposed a carbonated water corrosion model for Portland cement slurry, which agrees with experimental data. Cement has a relatively high degree of corrosion in acidic gas environments. In terms of building cement, the damage caused by corrosion can be reduced by adding anti-corrosion coatings or using other materials. However, in the case of oil well cement, the anti-corrosion performance can only be improved by adding additives. Polymer resin is a type of polymer material, and experimental research has found that adding a certain amount of polymer resin can effectively enhance the anti-corrosion ability of cement slurry. Peng et al. [8] studied the use of water-based modified epoxy resin to improve the corrosion resistance of oil well cement, and the study showed that the corrosion resistance of cement paste with the addition of modified epoxy resin was significantly improved. Qu et al. [9] studied the chlorine corrosion resistance of superhydrophobic anion exchange resin cement mortar in chloride environments. Resin anti-corrosion cement mortar is expected to extend the service life of reinforced concrete in chloride salt environments. Tan et al. [10] considered using organic anti-corrosion resin composite anti-corrosion agents to improve the corrosion resistance of cement slurry. Using slag and resin as anti-corrosion agents can effectively reduce the degree of corrosion in cement slurry. Guo et al. [11] used four different dosages of water-based epoxy resin as polymer cement repair materials. The flexural toughness of epoxy resin-modified OPC mortar is significantly improved, and the compressive strength is preserved. Aguiar et al. [12] used epoxy resin surface treatment to improve the carbonation resistance of concrete. Epoxy resin exhibits better performance than siloxane resin. Resin-based polymers are compatible with water, and their application in cement slurry anti-corrosion has great application value. However, there is currently limited research on the long-term anti-corrosion performance of polymer resin on cement slurry for well cementing. Studying the impact of polymer resin on the long-term corrosion performance of cement slurry is of great significance for the application of polymer resin in acidic and complex gas wells and geological storage wells.

Currently, the evaluation of the corrosion degree of cement slurry is mainly based on indoor experiments. Due to the long cycle and complex conditions of corrosion experiments, it is challenging to study the long-term corrosiveness of cement, observe the long-term corrosion changes in cement, and understand the long-term corrosion resistance of cement systems. Prediction is an effective method for predicting long-term changes based on early patterns. Regression models are commonly used to predict changes in cement properties, but their prediction accuracy could be higher. Finding a prediction method with higher accuracy and better response to cement corrosion changes is significant for oil and gas well development and oil well corrosion evaluation under acidic conditions.

With the development of computer technology, neural network algorithms have been widely applied to complex nonlinear systems based on human brain structures [13,14]. Hao et al. and Shiratori et al. [15,16] proposed an improved prediction model based on neural networks for simultaneously predicting electricity consumption and coal consumption during cement calcination, which has high accuracy. Terzic et al. [17] validated the optimal output of mechanochemical activation of pyrophyllite using an artificial neural network model with high prediction accuracy. Tsamatsoulis et al. [18] established an ANN model to predict changes in cement strength. The optimized prediction model has high accuracy and can be well applied in practice. Brown et al. [19] highlighted some recent effects to connect the ML and nanoscience communities and discussed the challenges and opportunities of artificial intelligence in nanoscience. Neural network models are widely used in complex problems and can better reflect actual changes. However, the BP model has a low convergence speed, is prone to falling into local minimization, and has low accuracy in long-term prediction [20]. Clustering particle algorithms can utilize individuals in a population to obtain the optimal solution, typically used to optimize neural network models [21,22,23]. However, the application of artificial intelligence in oil well cement corrosion is relatively limited, and there are still significant difficulties in establishing more accurate models based on actual situations.

In order to study the corrosiveness of polymer resin-modified oil well cement in acidic gas wells and evaluate the long-term corrosion degree of polymer anti-corrosion cement slurry, experimental research was conducted on the corrosion changes in polymer anti-corrosion cement slurry in corrosive environments. A corrosion prediction model based on the BP network was established by selecting three main influencing factors: corrosion temperature, pressure CO_2_, corrosion time, and corrosion depth as model parameters. The BP model was optimized using a particle swarm optimization algorithm and compared with regression models and traditional BP network models to verify the correctness of the corrosion prediction model. The long-term corrosion depth of polymer anti-corrosion cement slurry was predicted, and the long-term corrosion resistance of polymer anti-corrosion cement slurry was evaluated. The research results can be used to evaluate the long-term corrosion resistance of polymer anti-corrosion cement slurry in complex acidic gas wells.

## 2. Materials and Methods

### 2.1. Materials

Grade-G oil well cement was used as the primary material to prepare cement samples purchased from China Gezhouba Special Cement Factory. The chemical composition of the oil well cement is shown in Table 1. The filtrate reducer, retarder, dispersant, and reinforcer were used to adjust the performances of the oil well cement. The products were purchased from Jingzhou Jiahua Technology Co., Ltd. (Jingzhou, China). The reinforcer and resin were produced in the laboratory.

The filtrate reducer is one of the AMPS-type water-soluble polymers that can be used to reduce the water loss of the oil well cement slurry. The dispersant is an aldehyde ketone condensation polymer that is mainly used to adjust the surface charge of the cement particles to obtain cement slurry with appropriate rheological properties. The retarder consists of polymers with carboxylic and sulfonic acid groups that were mainly used to regulate the thickening time of cement slurry. Polymer resin was obtained from the laboratory and is a bisphenol A-type epoxy resin, which is mainly used to improve the corrosion resistance of cement. The solid phase accounts for 50% of resin materials.

### 2.2. Methods

#### 2.2.1. Preparation of Cement Samples

The preparation of cement slurry is in accordance with the provisions of the Chinese standard GB/T 19139–2012 [24]. The cement and various additives were mixed uniformly at 4000 rpm by a constant speed mixer (TG-3060A, Shenyang Tiger Petroleum Instrument Equipment Co., Ltd., Shenyang, China) according to the proportion of the experimental formula. The components of cement samples are shown in Table 2.

The prepared cement slurry was placed in a cylindrical mold with a height of 25.4 mm and a diameter of 25.4 mm. Then, the mold was placed in a 70 °C water bath for constant temperature curing for 24 h to form an uncorroded solidified cement sample.

#### 2.2.2. Corrosion Simulation Experiment

**Figure 1:**
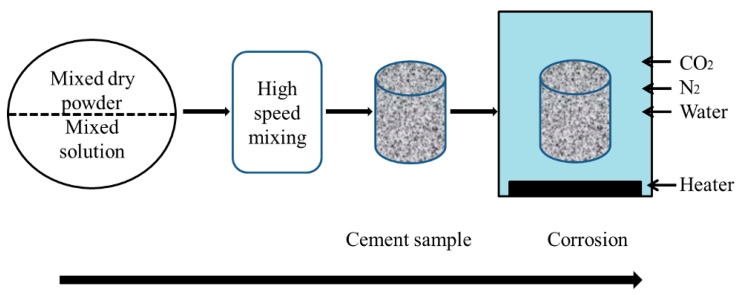
The simulation diagram of oil well cement corrosion.

A high-temperature and high-pressure corrosion tester (TG-7370D, Shenyang Taige Petroleum Instrument Equipment Co., Ltd., Shenyang, China) was set according to the conditions in the experimental scheme design (Table 3), and the uncorroded cement sample was cured in the high-temperature and high-pressure corrosion tester for the corrosion simulation test. Corrosion depth and compressive strength were selected as the observations [25]. The simulation diagram of oil well cement corrosion is shown in Figure 1. In the experiment, the temperature and pressure of CO_2_ changes are controlled according to the range of experimental parameters, respectively, and different times and conditions are obtained. The corrosion depth is determined according to the testing method in Section 2.2.3.

#### 2.2.3. Measurement of Corrosion Degree

**Figure 2:**
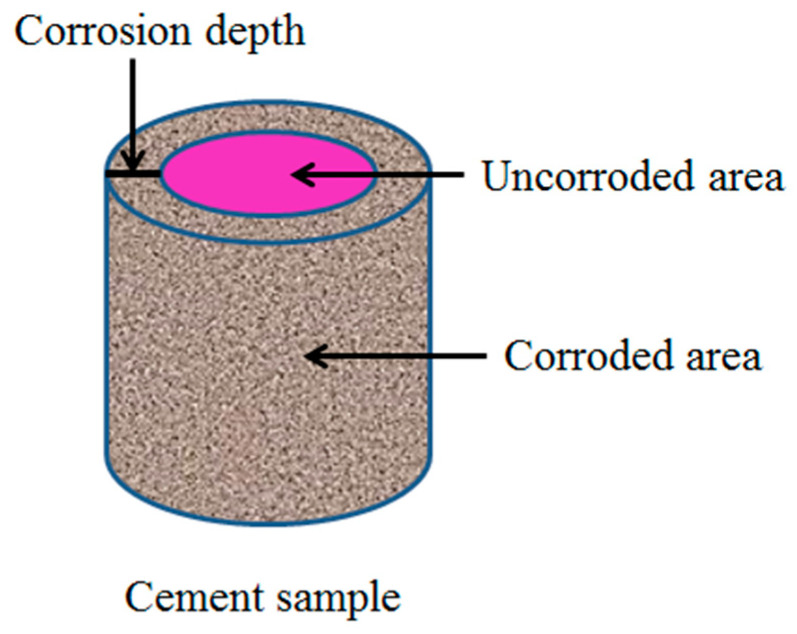
Corrosion of cement sample.

The corrosion area was calibrated with the characteristic of phenolphthalein turning red when encountering alkali. The four boundary thickness values of the sample that did not turn red were measured with a vernier caliper, and the average value was taken as the corrosion depth of the cement sample [26]. As shown in Figure 2.

The calculation of corrosion degree is

*P_c_* = *h*/*r* × 100%

where $P_{c}$ is the corrosion degree of oil well cement; *h* is the corrosion depth; and *r* is the radius of the oil well cement sample, which is 12.7 mm in this article.

#### 2.2.4. Test of Compressive Strength

The performance testing of the cement slurry was carried out in accordance with the provisions of the Chinese standard GB/T 19139–2012 [24]. The cement sample was removed from the mold and placed under the universal testing machine for a uniaxial compression test after the surface was wiped to test the compressive strength [27,28]. The universal mechanical testing machine (HY-20080, Shanghai Hengyi Precision Instrument Co., Ltd., Shanghai, China) was used to evaluate the compressive strength of the cement sample. Compressive strength is the maximum stress of the cement slurry in the process of compression failure. The sample used was a cylinder with a diameter of 25.4 mm and a height of 25.4 mm. Three cement stones were tested in each group, and the average value was calculated as the experimental result.

The calculation of compressive strength is

*P = F/A*

where *P* is the compressive strength of the sample, *F* is the load force, and *A* is the bearing area of the sample.

#### 2.2.5. Analysis of Micromorphology

The cement sample was dried in the oven at 60 °C for 24 h after the hydration was terminated. The surface morphology of the smooth trim sample was observed using the emission field scanning electron microscope (SEM). The prepared cement sample was crushed, and the smooth part in the middle was taken as the sample. The samples are dried in an oven, and the microscopic morphologies of the cement samples are observed using a scanning electron microscope (SU8010, Hitachi, Tokyo, Japan).

#### 2.2.6. PSO Algorithm

**Figure 3:**
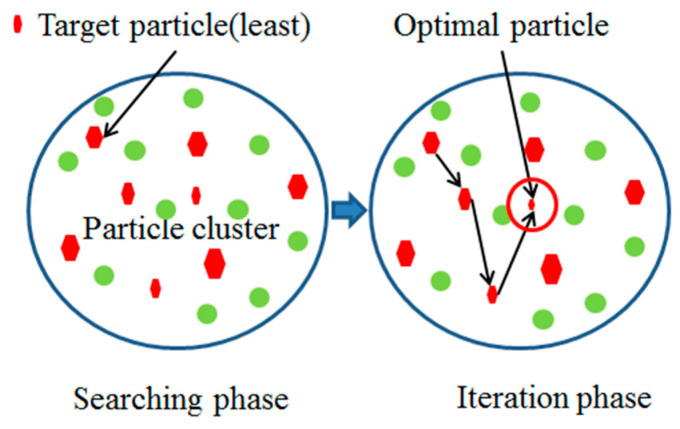
PSO schematic diagram.

Particle swarm optimization is based on the bird swarm search. The optimization problem was set as particles, the potential solution in the D dimension space of the particle was searched, the fitness function judged the potential solution, the optimal solution was updated by iterative search, and the running stopped when the maximum number of iterations or the best potential solution is found in the particle swarm optimization algorithm [29,30]. The PSO principle is shown in Figure 3.

### 2.3. Model Simulation

**Figure 4:**
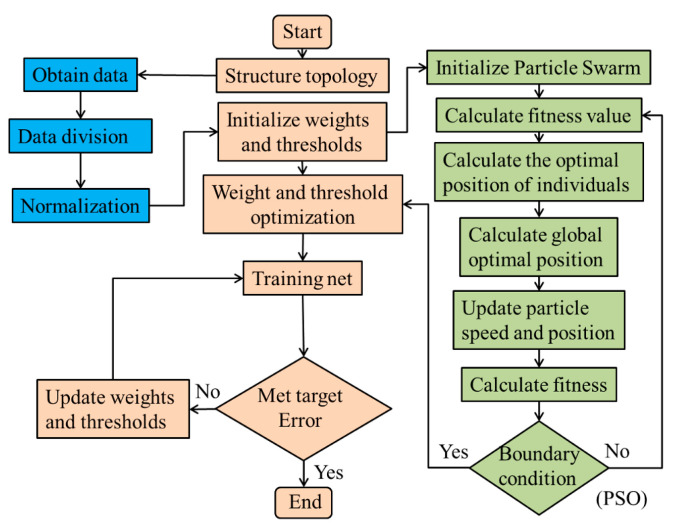
PSO-BP algorithm flow chart.

The neural numbers of hidden layers were selected as 3, 7, 10, and 12, respectively, and the three-layer BP neural network of 3-3-1, 3-7-1, 3-10-1, and 3-12-1 was established in the MATLAB 2021a software. The PSO algorithm was used to optimize the initial weights and thresholds of the BP neural network. The network algorithm flow chart is shown in Figure 4.

The number of hidden layer neurons h was selected according to the following formula [31,32].

$$H=\frac{\left(m+n\right)}{2}+\alpha$$

where *m* is the number of neurons in the input layer, *n* is the number of neurons in the output layer, and *α* is the random value in [0, 10].

The normalization formula was used to standardize the sample data. The calculation formula is as follows [33,34]:

$$x=\frac{x_{i}-x_{min}}{x_{max}-x_{min}}$$

where *x* is the normalization result and $x_{max}$ and $x_{min}$ are the maximum and minimum values of the data, respectively.

The error between the output and the actual value is

$$E_{i}=O_{i}-y_{i}$$

The overall mean square error (MSE) of the network output is

$$E_{t}=\frac{1}{n}\sum_{i=1}^{n}{{(O}_{i}-y_{i})}^{2}$$

where *i* is the number of trainings; $O_{i}$ is the network output value of the ith training; $y_{i}$ is the actual value of the sample for *i*-th training; and *N* is the number of training samples.

A total of 100 sample groups were obtained from the experiment. An amount of 80 groups were used as training samples, 10 groups as validation samples, and 10 groups as test samples. The target error and the maximum iterations are selected as the boundary conditions. The parameters of the prediction model are shown in Table 4.

## 3. Results

### 3.1. The Effect of Polymer Resin on the Properties of Oil Well Cement

**Figure 5:**
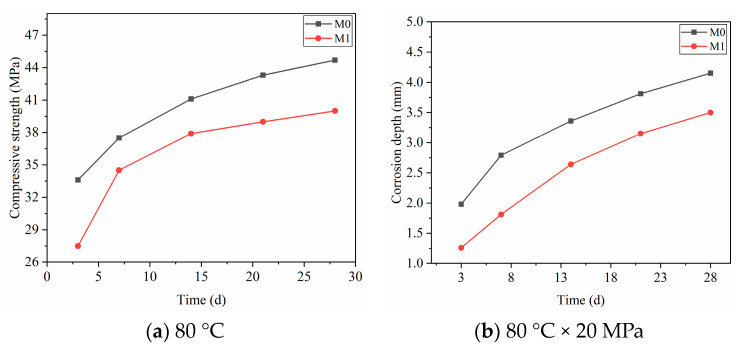
The influence of polymer resin on the compressive strength and anti-corrosion performance of cement: (**a**) the effect of resin on the compressive strength; (**b**) the effect of resin on the corrosion depth.

Figure 5 shows the effect of polymer resin on the compressive strength and corrosion resistance of oil well cement. In the corrosion process, the condition is 80 °C × 20 MPa. After adding a certain amount of polymer resin, the strength of the cement slurry decreases to a certain extent. However, the corrosion depth of cement paste under acidic CO_2_ conditions is significantly reduced compared to that of blank cement paste. When the amount of polymer resin added is 10%, oil well cement still has high compressive strength and excellent corrosion resistance. Resin polymers are dispersed in the cement slurry, forming a polymer film that blocks the connection between hydration products and reduces the strength of the cement. However, at the same time, polymer films protect cement hydration products from acidic gas corrosion, enhancing their corrosion resistance.

### 3.2. Corrosion Law of Polymer Anti-Corrosion Cement Slurry

**Figure 6:**
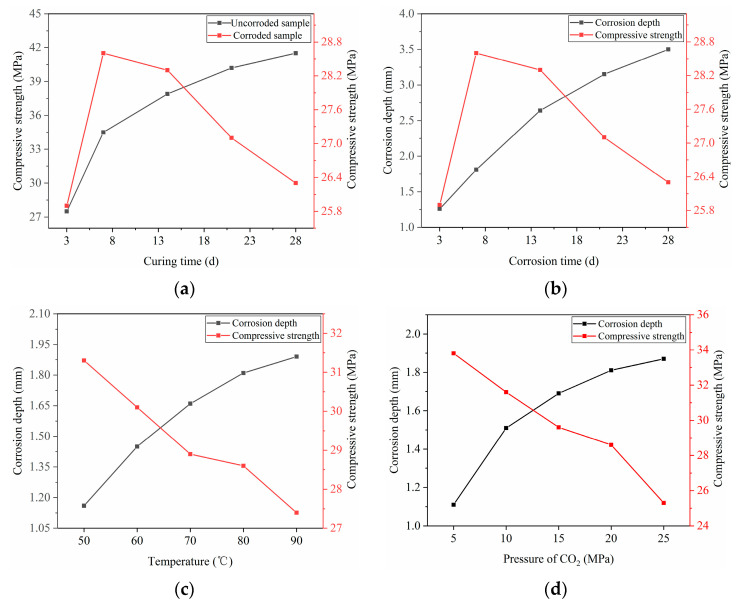
Changes in polymer anti-corrosion cement under corrosive conditions: (**a**) 80 °C, (**b**) 80 °C × 20 MPa, (**c**) 20 MPa × 7 d, (**d**) 7 d × 80 °C.

The corrosion changes in polymer anti-corrosion cement slurry under CO_2_ acidic conditions were experimentally studied, and the experimental results are shown in Figure 6.

In Figure 6a, the 7-day compressive strength of polymer anti-corrosion cement is 5.9 MPa less than that of uncorroded cement samples. As shown in Figure 6b, the corrosion depth and corrosion time increase, and the compressive strength decreases after a period of growth with the increase in corrosion time. According to Figure 6c,d, the corrosion depth and compressive strength of polymer anti-corrosion cement increase in the corrosive environment with the increase in corrosion time, corrosion temperature, and pressure of CO_2_.

### 3.3. Establishment of Corrosion Prediction Model

Four PSO-BP neural networks with a different number of hidden layer neurons were constructed based on the experimental data and network structure topology. The test samples were trained in the network and compared with the actual value [35]. The appropriate network structure is selected via error verification. The experimental results are as follows.

The results in Table 5 show that the established prediction model has high accuracy. The PSO-BPNN1 and PSO-BPNN2 networks have significant testing errors, resulting in significant deviations between the test and actual values.

### 3.4. Evaluation of Long-Term Corrosion Degree of Polymer Anti-Corrosion Cement

#### 3.4.1. Prediction of Corrosion Depth

In order to study the applicability of the PSO-BP model in predicting the long-term corrosion of cement, REG, BPNN, and PSO-BPNN3 are used to predict the long-term corrosion depth, respectively. The prediction results are as follows.

Table 6 shows that the predicted values of the traditional BPNN model are relatively small compared to the regression model, and their change trend is very small, possibly falling into local minima. The predicted values based on the PSO-BPNN3 model are generally close to the regression model, indicating no deviation from reality. Regression models are based on empirical formulas for prediction, and their predicted values have high reliability, but their accuracy is relatively low. The PSO-BPNN3 model has high accuracy, and its predicted values can better reflect the actual changes in long-term corrosion of cementing cement.

#### 3.4.2. Evaluation of Long-Term Corrosion Degree

In order to study the corrosion degree of cement slurry in long-term corrosive environments, based on a prediction model, the long-term corrosion prediction depth and corrosion degree calculation formula were used to evaluate the long-term corrosion degree of cement sample M0 without adding polymer resin and cement sample M1 with adding 10% polymer resin.

**Figure 7:**
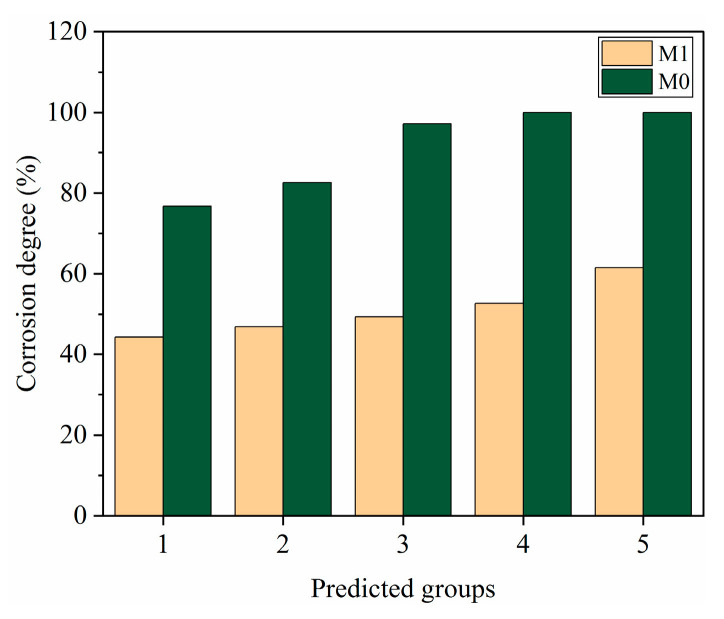
Evaluation of long-term corrosion degree of oil well cement.

From Figure 7, it can be seen that based on the experimental anti-corrosion cement slurry system, under corrosion condition 1, when the cement stone M1 is corroded for 365 days, the corrosion degree is 43.6%, and the corrosion degree of M0 is 76.69%. Under corrosion condition 2, after 365 days of corrosion, the corrosion degree of cement stone M1 is 46.2%. The corrosion degree of the cement sheath is less than 50%, and the structure of the cement sheath is damaged due to corrosion but still has a certain degree of integrity. The corrosion degree of M0 is 82.55%, and the corrosion degree is greater than 50%, which has been significantly corroded. Under corrosion conditions 4 and 5, when the cement stone is corroded for 1500 days, the corrosion degree of M1 is 52.66% and 61.52%, but the corrosion degree of M0 is 100%. The sample M1 with polymer resin added still maintains good structural integrity, while the cement sample M0 without resin addition has completely corroded. In long-term corrosion, the depth and degree of corrosion of the cement sheath continue to increase, and its dense structure is damaged, seriously affecting its integrity. The cement slurry added with polymer resin has a relatively lower corrosion degree and good corrosion resistance. By predicting the corrosion degree of cement under different corrosion conditions and times, the long-term sealing ability of the cement sheath can be evaluated, guiding the anti-corrosion design of cement slurry.

## 4. Discussion

### 4.1. Anti-Corrosive Mechanism of Polymer Resin

**Figure 8:**
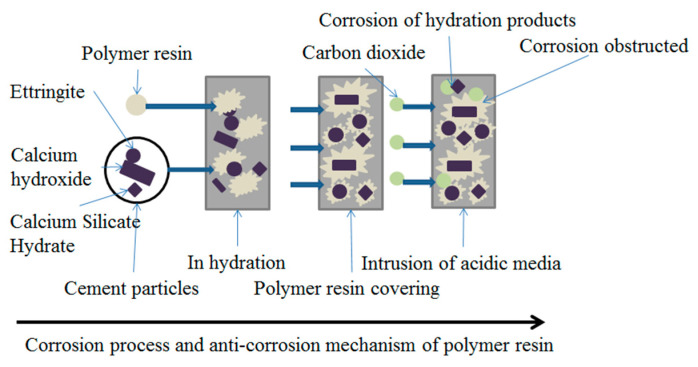
Schematic diagram of resin anti-corrosion mechanism.

The anti-corrosion mechanism of polymer resin in oil well cement is shown in Figure 8. Polymer resin is dispersed in the cement slurry, forming a polymer film encapsulating hydration products. When an acidic carbon dioxide gas medium invades the interior of cement slurry, the hydration products are corroded and damaged by the acidic medium, leading to the structural damage of cement stone. The hydration products wrapped in polymer resin films can effectively block acidic media and protect cement hydration products from damage. At the same time, the resin film encapsulates or fills between the hydration products, improving the overall density of the cement stone, reducing the invasion of CO_2_, and reducing the erosion degree of acidic media on the cement [36].

### 4.2. PSO-BP Model

**Figure 9:**
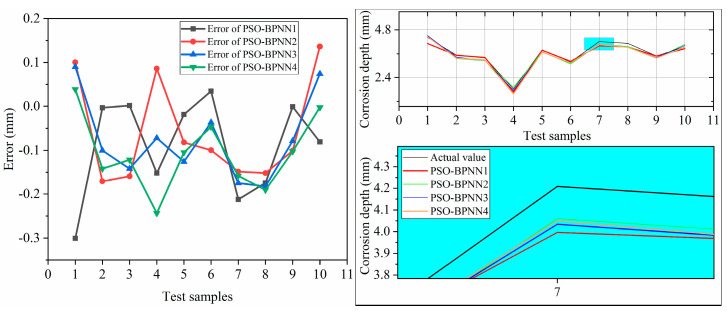
Test error of network with a different number of hidden layer nodes.

The results in Figure 9 show that the established prediction model has high accuracy. The PSO-BPNN1 and PSO-BPNN2 networks have significant testing errors, resulting in significant deviations between the test and actual values. The number of network nodes cannot effectively reflect the mapping relationship between input and output, ignoring the characteristics of certain sample data and resulting in significant errors. The error of PSO-BPNN4 is also relatively large, with good fitting at some data points, but there are cases where there is a significant deviation at some data points. The PSO-BPNN3 has the smallest testing error and a slight difference between the predicted and actual values, which can better reflect the actual situation. When there are fewer hidden layer nodes, it is easy to cause overfitting. When new sample data is input, the mapping results in output values that do not match the actual situation, resulting in significant errors in the network [37,38]. According to the results, PSO-BPNN3 has high accuracy and can be used for predicting the corrosion depth of polymer anti-corrosion cement.

### 4.3. Comparison between the PSO-BP Model and the Traditional BP Model

**Figure 10:**
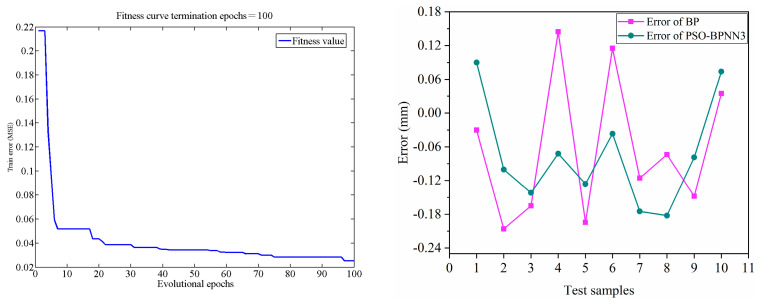
Comparison between the PSO-BP Network and the traditional BP Network.

In order to analyze the differences between the PSO-optimized model and traditional BP model. A traditional BP model (BPNN) was constructed (3-10-1) and compared with the PSO-BP model. The prediction was conducted with test sample data after the training. The test errors of the two models were compared. The experimental results are as follows. It was shown in Table 7 and Figure 10 that the PSO-BP neural network has higher precision, faster network convergence speed, a smaller error of test sample data, lower deviation, and higher stability and reliability than the traditional BP model.

### 4.4. Comparison between the PSO-BP Model and the Regression Model

An empirical formula-based regression model (REG) was obtained based on experimental data as follows [20]:

$$h=2.354\times \ln t+0.0182\times T+0.2693\times \sqrt{P_{{CO}_{2}}}-2.775$$

where h is the corrosion depth; *t* is the corrosion time; *T* is the corrosion temperature; and $\sqrt{P_{{CO}_{2}}}$ is the pressure of CO_2_.

The R^2^ of the fitting function is 0.9746, and the mean square error (MSE) of the test samples is 0.0339.

**Figure 11:**
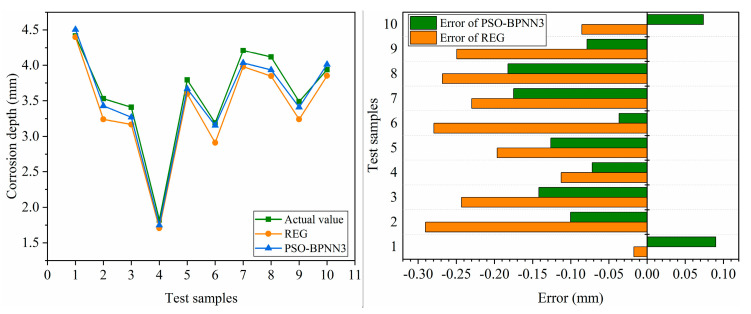
Comparison between the PSO-BP model and the regression model (REG).

To analyze the difference in accuracy between the PSO model and regression model in predicting the corrosion of polymer anti-corrosion cement, The PSO-BPNN3 and REG were used to predict the corrosion depth, respectively, and the results were compared. The experimental results are as follows. From the results in Figure 11, the accuracy of the regression model based on empirical formulas is relatively low. The BP neural network model optimized using PSO has higher accuracy compared to multiple regression models.

### 4.5. Micromorphology of Cement Samples

**Figure 12:**
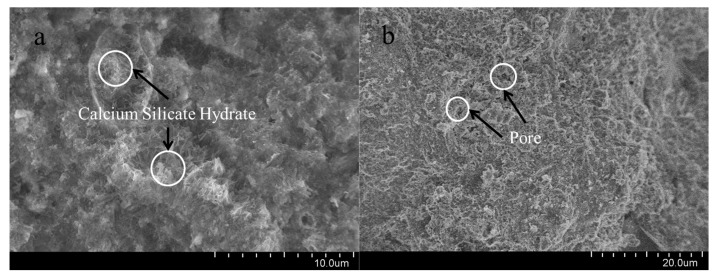
SEM of cement samples (the pressure in the condition is the pressure of CO_2_). (**a**): Uncorroded cement sample (7 d × 80 °C). (**b**): Corroded cement sample (7 d × 80 °C × 20 MPa).

In order to analyze the microstructure changes in oil well cement under corrosion conditions, the micro-morphology of oil well cement before and after corrosion was observed using a scanning electron microscope. The scanning electron microscope diagram is shown in Figure 12.

Figure 12b shows that the corroded cement sample has more pores, and compared to the pre-corroded cement sample, the structure of the cement sample is relatively loose. The dense structure of the cement sample is destroyed, reducing its strength in corrosive environments. In Figure 12a, there are no obvious corrosion pores inside the cement stone added with polymer resin, and there are many hydrated calcium silicate and calcium hydroxide crystals wrapped in polymer films with a dense overall structure [39]. By comparing the microstructure of two types of cement stone after corrosion, it is further indicated that under acidic conditions, polymer resin can effectively improve the corrosion resistance of cement stone and ensure its integrity.

## 5. Conclusions

(1) Adding polymer resin can reduce the strength of cement to some extent but greatly enhance the corrosion resistance of oil well cement. Polymer anti-corrosion cement has lower corrosion than blank cement slurry in long-term corrosion.
(2) The PSO algorithm can accelerate the convergence of BP neural networks and avoid BP neural networks falling into the local extremum. The PSO-BP model has higher accuracy in predicting the long-term corrosion depth of cement in complex geological environments compared to regression models and traditional BP networks.
(3) The artificial neural network prediction model based on PSO optimization performs well in predicting and evaluating the long-term corrosion degree of polymer anti-corrosion cement and can guide the design and evaluation of anti-corrosion cement slurry.

## Figures and Tables

**Table 1:** Chemical composition of G-class oil well cement.

Component	CaO	SiO_2_	Fe_2_O_3_	Al_2_O_3_	MgO	Na_2_O + K_2_	Others
Content (%)	64.2	22.5	4.4	4.1	1.6	0.38	2.82

**Table 2:** Components of cement slurry.

Samples	Cement	Water	Reinforcer	Dispersant	Filtrate Reducer	Retarder	Resin
M0	100	44	2	0.5	2	0.4	0
M1	100	39	2	0.5	2	0.4	10

**Table 3:** Experimental scheme.

Sample Groups	Factors and Control Scope			Observations	
	Time (d)	Temperature (°C)	Pressure of CO_2_ (MPa)	Corrosion Depth (mm)	Compressive Strength (MPa)
100	1~60	50~90	5~25	h	P

**Table 4:** PSO-BP model parameter settings.

Variable	Parameter		
Input parameter	Pressure of CO_2_, Temperature, Time		
Network structure	3-3-1, 3-7-1, 3-10-1, 3-12-1		
Variable	Parameter	Variable	Parameter
Total number of samples	100	Output parameters	Corrosion depth
Training methods	Gradient descent (L-M)	Particle dimension	16, 36, 51, 61
Number of training samples	80	Learning rate of BP	0.01
Weight correction method	Error backpropagation	Number of test samples	10
Maximum iterations of BP	2000	Inertial factor	0.7
Number of hidden layers	1	Particle swarm size	100
Maximum iterations of PSO	100	Target error (MSE)	0.001
Number of validation samples	10	Minimum position	−1
Learning parameters of PSO	0.05	Maximum position	1
Minimum speed	−0.1	Maximum speed	0.1

**Table 5:** Training results of different PSO-BPNN network structures.

Model	Structure	R^2^	Best Validation Error (MSE)	Iterations	Test Error (MSE)
PSO-BPNN1	3-3-1	0.9880	0.0024	19	0.0197
PSO-BPNN2	3-7-1	0.9955	0.0062	14	0.0163
PSO-BPNN3	3-10-1	0.9970	0.0022	4	0.0136
PSO-BPNN4	3-12-1	0.9953	0.0007	19	0.0216

**Table 6:** Prediction of long-term corrosion of oil well cement.

NO.	Corrosive Conditions			Prediction of Corrosion Depth (mm)		
	Pressure of CO_2_ (MPa)	Temperature (°C)	Time (d)	REG	BPNN	PSO-BPNN3
1	25	50	365	5.533	4.070	5.543
2	15	80	365	5.775	4.072	5.867
3	5	90	500	5.838	4.834	6.174
4	15	70	1000	6.624	5.358	6.583
5	20	80	1500	7.381	5.569	7.691

**Table 7:** Training results of PSO-BPNN3 and BPNN.

Model	Structure	R^2^	Best Validation Error (MSE)	Iterations	Test Error (MSE)
PSO-BPNN3	3-10-1	0.9970	0.0022	4	0.0136
BPNN	3-10-1	0.9941	0.0028	14	0.0184

## Data Availability

Data will be available upon request.
